# The pathogenesis of *Hematodinium* spp. in decapod crustaceans – recent advances and unanswered questions

**DOI:** 10.1017/S0031182025100930

**Published:** 2025-10

**Authors:** Andrew F. Rowley

**Affiliations:** Biosciences, Faculty of Science and Engineering, Swansea University, Swansea, UK

**Keywords:** crabs, decapods, dinoflagellate, epidemiology, *Hematodinium*, histopathology, immune evasion, Norway lobster, pathobiology

## Abstract

The dinoflagellates *Hematodinium* spp. are important endoparasites of a wide range of decapod crustaceans from across the globe. High prevalences of infection have been reported particularly in decapods of commercial importance including crabs and some lobster species. While such infections usually result in their death, the dynamics of these differ widely depending on location, the genotype of *Hematodinium* and host. This review aims to explore the interaction between these parasites and their hosts with particular emphasis on the diversity of host range, methods of detection, impact on fisheries and how this parasite multiplies within hosts without causing any apparent cellular immune response. Emphasis is placed on evaluating the future directions required to solve key unanswered questions of this increasingly important parasite.

## Graphical abstract

**Figure:**
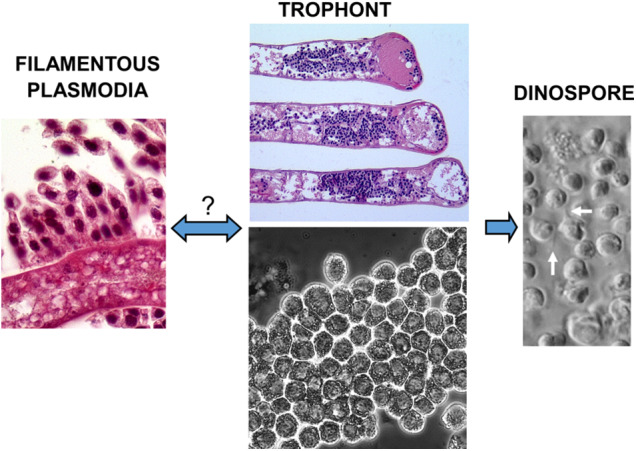

## Introduction and background

It is nearly a century since 2 French scientists, Chatton and Poisson (1931), recorded a novel protistan endoparasite in both shore crab (*Carcinus maenas*) and harbour crab (*Liocarcinus depurator*) haemolymph but at low prevalence (<1%). They named this parasite *Hematodinium perezi* after Professor Charles Pérez who had first observed it in shore crabs in 1905 and the drawings accompanying their paper showed several morphological forms including multinucleate elongate plasmodia with characteristic dino-mitotic chromosomes as seen in other dinoflagellates (Gornik *et al.*, 2019). Some 40 plus years later, the presence of *Hematodinium* sp. was reported in blue crabs, *Callinectes sapidus*, collected from several locations in North America but at a much higher prevalence (*ca*. 30%) at least in 1 site (Newman and Johnson, 1975). A second species of this parasite was identified in 1994 in Moreton Bay, Australia by Hudson and Shields (1994) in the sand crab, *Portunus pelagicus* and, based on its different morphology to the type species, it was named *Hematodinium australis*. Since these initial reports, over 45 species of marine decapod crustaceans from across the globe have been recorded as hosts to these parasites (see Alimin *et al.*, 2024; Small and Li, 2025 for maps of their geographic location). There are no known control measures or treatments to combat the effect of infections caused by *Hematodinium* spp. (Coates and Rowley, 2022; Small and Li, 2025).

**Figure 1.**
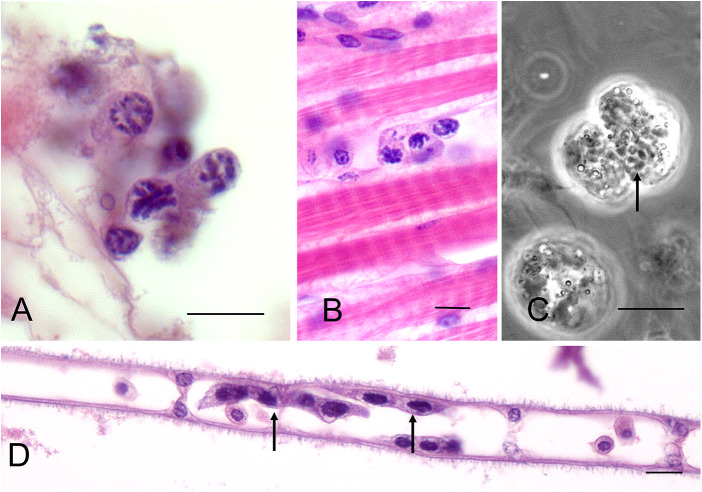
Examples of the variable morphology of life history stages of *Hematodinium*. (A) Characteristic condensed chromatin in individual trophonts in the hepatopancreas. (B) Trophonts in the connective muscle between muscle fibres. (C) Phase contrast view of live trophonts in the haemolymph showing condensed chromatin (arrow). (D) Multinucleate plasmodia (arrows) attached to the inner margin of gill lamella. Images A, B, and D, H&E-stained sections. Scale bars = 10 µm.

*Hematodinium* spp. belong to the eukaryotic phylum Dinoflagellata and members of this assemblage are found in both marine and freshwater environments. Most of the *ca*. 2400 representatives of this monophyletic group are free living plankton and are either photosynthetic autotrophs, mixotrophs or heterotrophs (Gómez, 2012). However, *ca*. 150 are ecto- or endo-parasites of a range of hosts including ciliates, invertebrates, vertebrates and other dinoflagellates (Coats, 1999). These parasitic forms include *Syndinium turbo* an endoparasite of various marine copepods (Skovgaard *et al.*, 2005) and the ectoparasitic *Amyloodinium ocellatum* found on various marine and brackish water fish where the trophont stage feeds on gill epithelia resulting in damage and inflammation (Rückert, 2025). The nucleus of dinoflagellates contains unusual DNA where the chromosomes are constantly condensed during interphase and this gives *Hematodinium* its characteristic nuclear morphology observed in live and fixed, stained preparations (Figure. 1A–D) making it relatively easy to identify these parasites in either haemolymph or solid tissues. The genome of many dinoflagellates is extremely large ranging from 1 to 250 Gbp (Lin, 2024) and in *Hematodinium* it is 4.8 Gbp (Gornik *et al.*, 2012), but the reason for this evolutionary expansion of the dinoflagellate genome is unclear (Talbert and Henikoff, 2012). A second unusual feature of the nuclear material of *Hematodinium* and many other dinoflagellates, is the apparent lack of histone proteins and their ‘replacement’ with novel DNA binding proteins christened dinoflagellate/viral nucleoproteins (Gornik *et al.*, 2011).

Many commercially important decapods from fisheries and aquaculture sectors are subject to parasitization by *Hematodinium* spp. including Norway lobsters (langoustines), *Nephrops norvegicus* (e.g. Field and Appleton, 1995; Appleton and Vickerman, 1998; Small *et al.*, 2006; Molto-Martin *et al.*, 2024; Martin *et al.*, 2025) and edible crabs, *Cancer pagurus* (Ní Chualáin *et al.*, 2009; Ní Chualáin and Robinson, 2011; Smith *et al.*, 2015) in Northern Europe, invasive blue crabs *C. sapidus* in the Mediterranean (Patrizia and Giorgio, 2018; Lattos *et al.*, 2024) and in their native range (Messick and Shields, 2000; Small *et al.*, 2019), Tanner, *Chionoecetes bairdi* (Meyers *et al.*, 1987; Wheeler *et al.*, 2007; Siddeek *et al.*, 2010), and snow crabs, *Chionoecetes opilio* (Taylor and Khan, 1995; Pestal *et al.*, 2003; Shields *et al.*, 2005, 2007; Fedewa *et al.*, 2025) in North America, and Chinese swimming crabs, *Portunus trituberculatus* (Li *et al.*, 2013; Wang *et al.*, 2017), mud crabs, *Scylla paramamosain* (Li *et al.*, 2008), giant tiger prawns, *Penaeus monodon* (Wang *et al.*, 2017), Ridgetail white prawns, *Exopalaemon carinicauda* (Xu *et al.*, 2010), Asian bush-clawed crabs, *Hemigrapsus takanoi* (Gong *et al.*, 2023) and mudflat crabs, *Helice tientsinensis* (Huang *et al.*, 2019) in China. The effect of disease by *Hematodinium* spp. on these populations can be profound and it is often fatal. In some, infections are enzootic within crustacean populations and apart from seasonal change in their occurrence, differ little from year to year in their presence. Such infections include those of langoustines in the Clyde Sea Area, Scotland, UK (Molto-Martin *et al.*, 2024) and edible crabs in South Wales, UK (Smith *et al.*, 2015). In others, including Tanner and snow crabs, environmental changes including increased water temperatures, appear to drive higher levels of infections both in terms of prevalence and severity (Table 1). Such epizootic outbreaks can alone result in population declines or exacerbate these caused by other factors such as overfishing and climate change. These can result in the closure of the fishery, economic loss due to the death of infected crabs, and to the loss of marketability, because of the bitter taste of the infected muscle in some crabs (hence the disease is sometimes referred to as bitter crab disease) (Balstad *et al.*, 2024).

**Table 1.** Examples of outbreaks of infections caused by *Hematodinium* in crustaceans of commercial importance

Host	Location	Fishery/aquaculture	Comments	References
Velvet swimming crabs (*Necora puber*)	Brittany, France	Fishery	96% decrease in crab capture believed to be at least partially driven by *Hematodinium*	Wilhelm and Mialhe (1996)
Blue crabs (*Callinectes sapidus*)	Atlantic and Gulf coasts, USA	Fishery	Decline in blue crab thought to be linked to disease including bitter crab disease caused by *Hematodinium* sp.	Messick and Shields (2000); Lee and Frischer (2004); Lycett and Pitula (2017)
Snow crabs (*Chionoecetes opilio*)	Newfoundland, Canada	Fishery	Epizootics driven by rising bottom temperatures (causing higher frequency of moulting and increased susceptibility to infection?)	Shields *et al.* (2005), (2007)
Snow (*C.opilio*) and Tanner (*C. bairdi*) crabs	East Bering Sea, Alaska, USA	Fishery	Declines in populations considered to be linked to crab mortality and recruitment failure. *Hematodinium* thought to be an important driver of mortality based on re-assessment of prevalence following improvement of detection assay (see Table 2)	Fedewa *et al.* (2025)
*Portunus trituberculatus* and *Penaeus monodon*	Various coastal regions of China including Shandong Peninsula	Aquaculture	High levels of mortality in polyculture pond system of *P. trituberculatus* and *P. monodon* caused by *H. perezi* (genotype II). Reservoirs of infection in co-located wild crabs with potential of transfer disease	Li *et al.* (2013); Wang *et al.* (2017); Huang *et al.* (2019)

The rationale for this review is not to simply update the existing excellent and comprehensive reviews of *Hematodinium* spp. published over the last 20 years (e.g. Stentiford and Shields, 2005; Small, 2012; Li *et al.*, 2021a; Alimin *et al.*, 2024) but to examine in depth some of the areas where our understanding of these infections is still uncertain. These include (1) an exploration of how these parasites can flourish in the tissues of so many different hosts apparently free of a cellular immune response, (2) the consequences of inadequate approaches to the detection of infected crustaceans of relevance to fisheries science and aquaculture development and (3) a reappraisal of infection dynamics and the involvement of secondary infections in the pathogenesis of these parasites taking into account the diversity of different parasite genotypes.

## How many species of *Hematodinium* are there?

While only 2 distinct species of *Hematodinium* have been described (*H. australis* and *H. perezi*) there is known diversity in the ITS region of rDNA within *H. perezi*. As reviewed by Small (2012), Li *et al.*, (2021a) and Small and Li (2025), there are 3 genotypes (I, II and III) in this latter species with differing host ranges and geographic locations. Genotype I, found mainly in Northern Europe, infects *L. depurator* and the shore crab, *Carcinus maenas* (Small and Li, 2025), while genotype II is chiefly restricted to China in a variety of crabs and shrimp (Xiao *et al.*, 2016; Li *et al.*, 2021a). *H. perezi* genotype III is found in North America in a wide range of crustaceans including *C. sapidus* (Pagenkopp Lohan *et al.*, 2012, 2013; Small and Li, 2025). Whether crustaceans are susceptible to infections with more than 1 genotype (i.e. infected simultaneously with several genotypes) is unknown.

## What is the host range of *Hematodinium* spp.?

There are still increasing numbers of reports year on year of new hosts shown to be subject to infections by *Hematodinium* spp. For instance, since a recent review in 2021 by Li and co-authors (Li *et al*., 2021a), several new hosts have been reported including Asian bush-clawed crabs, *Hemigrapsus takanoi* (Gong *et al.*, 2023) the marine crab, *Macrophthalmus abbreviatus* (Liu *et al.*, 2024) and the Chinese mitten crab, *Eriocheir sinesis* (Kerr *et al.*, 2025). This continued increase in host range suggests that all true (brachyuran) crabs in the marine environment are susceptible to this host-generalist parasite. Outside of these true crabs, king crabs (Ryazanova *et al.*, 2010, 2021) of the family Lithodidae, some penaeid shrimp (Wang *et al.*, 2017), the homarid, *N. norvegicus* (Field and Appleton, 1995; Small *et al.*, 2006; Molto-Martin *et al.*, 2024; Martin *et al.*, 2025), the anomuran hermit crab, *Pagurus bernardus* (Hamilton *et al.*, 2009) and possibly some amphipods (Pagenkopp Lohan *et al.*, 2012) are hosts. Notable by their absence as hosts are the commercially important clawed lobsters, *Homarus americanus* and *H. gammarus* found in North America and northern Europe, respectively. These 2 species have been subject to extensive investigations of their parasites and pathogens over the last few decades (see reviews by Davies and Wootton, 2018; Cawthorn, 2011 for details) yet no reports exist on the presence of *Hematodinium* or *Hematodinium*-like parasites in their tissues. A preliminary trial to infect juvenile *H. gammarus* with trophonts of *Hematodinium* sp. (probably *H. perezi* genotype I) from an infected edible crab, *C. pagurus* donor, failed to find any evidence of ensuing infection (Davies and Rowley, 2015). Therefore, it can be concluded that while all brachyuran crabs living in high-salinity environments are probably susceptible, this is not the case for all homarids. This lack of susceptibility in the latter group deserves further exploration to determine the mechanisms of resistance. Such an approach may shed light on how *Hematodinium* avoids detection and elimination in susceptible hosts.

Both the European shore (= green) crab, *C. maenas* and the blue crab, *C. sapidus* are recognized as ‘successful’ invasive non-native species. The ‘natural’ range of shore crabs is in northern Europe but they have spread to the Americas, Africa and Australia over the last 200 years (Ens *et al.*, 2022). Similarly, blue crabs, native to the Western Atlantic, appeared in the Mediterranean by the 1940s and the Atlantic coast off Morocco in 2019 (Oussellam *et al.*, 2023). When these non-native species relocate to new regions they can either bring parasites, such as *Hematodinium,* with them or act as novel hosts by spillback. There are reports of shore crabs infected with *Hematodinium* along their invasion route in the Faroe Isles but not in Nova Scotia in Canada (Bojko *et al.*, 2018). Similarly, infected blue crabs have been recorded in the Mediterranean (Lattos *et al.*, 2024) and off Morocco in the Atlantic Ocean (Lamkhalkhal *et al.*, 2024). Whether these infections were carried by infected crabs during these invasions or acquired from other native crabs once in their new locations, is unclear but this gives the potential for mixing of *Hematodinium* spp. genotypes as already described. The form of this parasite in blue crabs in the Mediterranean appears to be distinct to the *H. perezi* genotype III found in this species back in its native range in America (Lattos *et al.*, 2024; Small and Li, 2025) suggesting its acquisition from other decapods in its new environment. Similarly, the recent observation of *H. perezi* genotype 1 in non-native Chinese swimming crabs, *Eriocheir sinensis* in the River Thames, UK, suggests that it has become an accidental host for this parasite in its new habitat (i.e. by spillback) (Kerr *et al.*, 2025).

## How are *Hematodinium* infections identified?

There are several approaches to the detection of *Hematodinium* that vary in terms of their practicality, cost, sensitivity and specificity (Table 2). Visual approaches, such as those carried out onboard survey vessels or in the field, while lending themselves to rapid assessment, are not sensitive enough and only identify heavily affected animals in the later stages of infection (e.g. Beevers *et al.*, 2012; Fedewa *et al.*, 2025; Martin *et al.*, 2025). These approaches include the pleopod method used with langoustines (e.g. Field *et al.*, 1992; Field and Appleton, 1995; Albalat *et al.*, 2016; Molto-Martin *et al.*, 2024; Martin *et al.*, 2025) and colour/opacity changes in the appearance of the ventral carapace (e.g. Pestal *et al.*, 2003; Martin *et al.*, 2025) that can be subjective to the investigator. Indeed, a recent re-evaluation of seasonal prevalence data from the east Bering Sea in snow and Tanner crabs compared prevalence data of disease between a macroscopic assessment based of carapace colour change compared with the more sensitive PCR-based method (Fedewa *et al.*, 2025). Not only did the visual method fail to identify 93% of infected individuals but the study demonstrates a rapid rise in the prevalence of disease from just *ca*. 10% in 2015 to 40% by 2017 lending weight to the concept that *Hematodinium* infections are a major driver in population declines of these crabs (Tables 1 and 2).

**Table 2.** A comparison of the methods commonly used to determine the presence of *Hematodinium* in decapods and environmental samples

Method	Sensitivity*	Specificity*	Comments (‘*pros and cons’*)	Examples
Visual (colour change in carapace)	Low (only identifies advanced infections)	Medium	Rapid test particularly relevant to field observations but underestimates infection levels in animals with sub-clinical infections	Pestal *et al.* (2003)
Visual (e.g. pleopod observation in langoustines)	Low-medium (known to miss low level infections)	Medium	Rapid test, that usually involves use of low power microscope and looks for accumulations of parasites under the cuticle	Field *et al.* (1992); Molto-Martin *et al.* (2024); Martin *et al.* (2025)
Haemolymph opacity (examination by eye)	Low	Low	Milky appearance reflects haemolymph parasitaemia but many other infectious agents (e.g. fungi and haplosporidians) can also give this appearance. Needs follow-up with microscopy and/or PCR to prove presence of *Hematodinium*	Xu *et al.* (2010); Small (2012)
Haemolymph preparations	Medium-high	High	Needs experienced microscopist. Preparations can be observed with phase contrast or brightfield optics using neutral red to stain lipid droplets in parasites that aids in differentiation of parasites from haemocytes. Rapid assessment that is quantifiable	Messick (1995); Pestal *et al.* (2003); Shields (2017); Davies *et al.* (2019), Davies *et al.* (2022)
Histology	Medium-high	High	*Hematodinium* forms easily seen due to unusual nuclei with condensed chromatin that stain intensely blue/black with haematoxylin dye (Figure. 1). Advantage is that histology reveals the site and location of parasites and general pathology of infection. Lengthy procedure that needs careful interpretation to avoid artefacts of processing	Field and Appleton (1995); Stentiford *et al.* (2002); Wheeler *et al.* (2007); Ní Chualáin and Robinson (2011); Small *et al.* (2012); Davies *et al.* (2019)
Immunoassays (ELISA, blots, immunofluorescence)	Medium-high	High	Lengthy procedure relies on specificity of antiserum not commercially available. Rarely used after advent of PCR-based assays	Stentiford *et al.* (2001a); Small *et al.* (2002); Gornik *et al.* (2013)
PCR and qPCR (and ISH**)	High	High (may allow determination of genotypes I–III of *H. perezi*)	Several primer sets developed including long base pair sequence in ITS1&2 region of rRNA complex useful for taxonomic elucidation. ISH may be a useful addition to basic histology in some cases	Hudson and Adlard (1994, 1996); Gruebl *et al.* (2002); Small *et al.* (2006, (2007)); Xie *et al.* (2025)
eDNA	High	High	Primary employed to find free living forms of parasite in water column and those associated with zooplankton (e.g. megalopa stage in crab life cycle). Can be highly sensitive	Li *et al.* (2010); Hamilton *et al.* (2011); Hanif *et al.* (2013); Davies *et al.* (2022)

* Low/medium/high.

** In situ hybridization.

An increasing number of studies have deployed a comprehensive range of approaches to assess the importance of *Hematodinium* in their hosts. Some include histology that has the advantage of revealing changes in host tissues as these parasites multiply in the haemolymph but dealing with large numbers of samples for histology necessary to adequately monitor changes in seasonal prevalence and severity of infection, can be challenging. Using this approach, together with PCR-based methods, gives the researcher a full picture of this condition that PCR-based methods alone cannot accomplish. For instance, Davies *et al.*, (2019) carried out a large-scale survey of *Hematodinium* infections in the shore crab, *C. maenas* in South Wales, UK, over a 12-month period involving ∼1200 crabs. These were all initially examined with live haemolymph preparations to determine if crabs were infected followed up with histological analysis. All crabs regardless of infection status were also checked for *Hematodinium* using several sets of PCR primers (Small *et al.*, (2007); Gruebl *et al.*, 2002) specific for this parasite. Those crabs found to be positive by PCR but apparently negative by histology and microscopic examination of haemolymph, were re-reviewed by histology. This approach confirmed that histology alone correctly identified over 95% of all crabs with clinical and sub patent infections.

## How are *Hematodinium* spp. transmitted?

**Figure 2.**
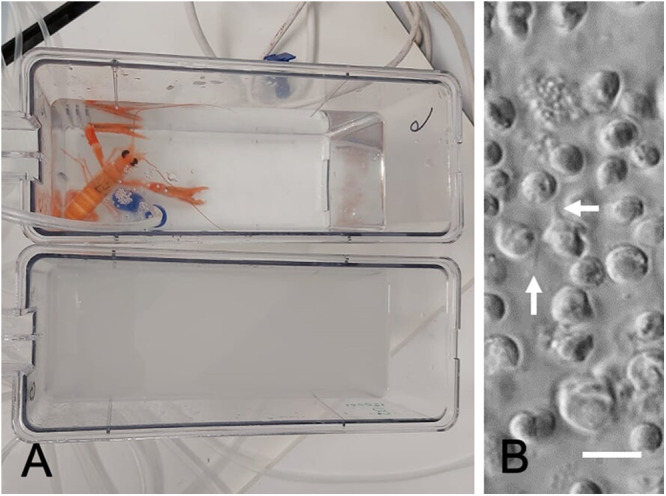
Dinospore formation and release in the Norway lobster, *Nephrops norvegicus*. (A) Aquarium-based release of clouds of dinospores from *N. Norvegicus*, note cloudy appearance of water post-release. (B) Appearance of flagellated (unlabelled arrows) dinospores using interference microscopy. Scale bar = 10 µm. Images courtesy of I. Molto-Martin and A. Albalat.

Late-stage infections in some crustaceans have been shown to generate a motile stage termed the dinospore (Figure. 2A,B) that develops in the haemolymph and is thought to be responsible for transmission of the disease (Chen *et al.*, 2023; Zhang *et al.*, 2025b). Two forms of dinospore have been described, termed micro- and macro- at least in some hosts (e.g. Appleton and Vickerman, 1998; Huchin-Mian *et al.*, 2017; Zhang *et al.*, 2025b). Microdinospores appear to be faster swimmers than the macro forms (Huchin-Mian *et al.*, 2017; Zhang *et al.*, 2025b). Therefore, macrospores may be less infectious because of their lower swimming activity (Chen *et al.*, 2023). Whether the dinospores of different genotypes of *H. perezi* have divergent abilities to swim and infect is unknown.

As there may be several species of susceptible decapods in the vicinity when a ‘cloud’ of dinospores is released from the gills, there is chance for the condition to be spread into other adjacent populations. The dinospore stage is relatively short-lived (>a few hours) and it is not tolerant of low salinity environments (Coffey *et al.*, 2012; Zhang *et al.*, 2025b). Its production is also stimulated by high water temperatures (Chen *et al.*, 2023). How dinospores gain access to the internal tissues of their hosts is unknown. Possible routes of entry include across the gut wall, via the gills with their thin outer epithelium and acellular cuticle, or across the main cuticle perhaps where there are injuries (Rowley and Coates, 2023). Transmission via cannibalism of infected animals has not been proven (Li *et al.*, 2011) probably because the stages of parasite observed in tissues (i.e. trophonts and filamentous trophonts/plasmodia) are not the natural invasive stage. However, intrahaemocoelic injection of trophonts taken from infected decapods will develop in naïve hosts and this has formed the basis of experimental infections aimed to investigate how these parasites develop in their hosts (e.g. Messick and Shields, 2000; Smith and Rowley, 2015). Natural transmission of infections between co-inhabitants have been observed under aquarium conditions using juvenile blue crabs (*Callinectes sapidus*) as experimental hosts (Chen *et al.*, 2023). Changes in temperature and high temperatures (25 ºC) were reported to favour dinospore production and transmission, and this was found to correlate with sea temperatures during peak transmission of the disease as found in the late summer – early autumn months in the USA. In contrast, peak transmission of *Hematodinium* sp. (probably *H. perezi* genotype I) in *C. pagurus* and *C. maenas* occurs in the winter months at low sea water temperatures (Smith *et al.*, 2015; Davies *et al.*, 2019) perhaps reflecting differences in the triggers for dinospore generation of *H. perezi* genotype III in the USA with genotype I in northern Europe.

## What are the effects of parasitization of crustaceans by *Hematodinium* spp.?

**Figure 3.**
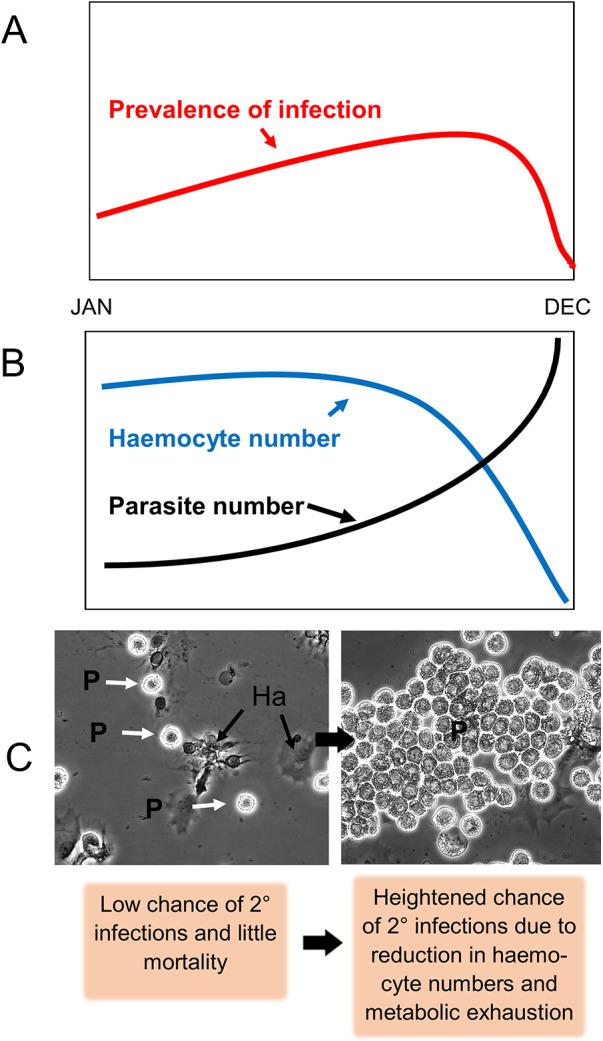
Schematic of infection dynamics of *Hematodinium* sp. (probably *H. perezi* genotype I) in the edible crab, *Cancer pagurus* based on the results in Smith *et al.* (2015) and Smith and Rowley (2015). (A) Change in prevalence of infection. (B) Seasonal changes in numbers of haemocytes and *Hematodinium* sp. (C) Phase contrast micrographs of low to high grade infections (left to right panels) in the haemolymph with *Hematodinium* sp. (P). Note adherent haemocytes (Ha) in low grade infection and their absence in high grade infection in the right-hand panel. This model of infection dynamics may differ in the other genotypes of *H. perezi* II and III and the prevailing environmental conditions including temperature and salinity.

While it is widely reported that decapods parasitized by *Hematodinium* die probably because of the release of dinospores causing damage to the gills and metabolic exhaustion, the timescale of this from initial infection through to death varies widely between host, parasite genotype and their geographic location. Generally, crustaceans in warmer waters appear to succumb to parasitization quicker than those in colder waters probably because multiplication of *Hematodinium* is temperature dependent (e.g. Huchin-Mian *et al.*, 2018; Shields, 2019) and so changes in sea water temperature caused by climate change may in future affect the distribution and importance of these parasites in some locations (Byers, 2020; Rowley *et al.*, 2024). Physiological and biochemical studies reveal that infected hosts have reduced glycogen reserves in the hepatopancreas, and glucose and protein in their haemolymph (Table 3 and references therein). Moulting is inhibited by an unknown mechanism hence increasing the intermoult period at least in some crabs (Smith and Rowley, 2015). A major change of significance to immune responsiveness and haemolymph clotting is the marked reduction in the number of circulating haemocytes (termed haemocytopenia) in several host species (Conneely and Coates, 2025). The cause of this rapid reduction in haemocyte numbers is unknown but could result from a targeting of haemopoiesis or simply due to metabolic exhaustion resulting in shifts in energy resources away from haemocyte production. In edible crabs (*C. pagurus*) where the time from first infection to death under aquarium conditions is lengthy (93–378 days post-challenge in *C. pagurus*; Smith and Rowley, 2015), there appears to be 2 phases in haemocytopenia (Figure. 3A–C). Initially, the numbers of haemocytes in infected crabs are relatively unchanged but as the parasites in the tissues quickly develop in warmer months, haemocyte numbers can rapidly decline at least in some infected animals (Smith and Rowley, 2015). This terminal decline in haemocyte numbers leaves edible crabs susceptible to secondary infections including that caused by the fungus, *Ophiocordyceps* (Stentiford *et al.*, 2003; Smith *et al.*, 2013) such that these animals may die from a combination of metabolic exhaustion, the production and release of dinospores via the gills and/or secondary infections. It is important to stress that to the author’s knowledge, such secondary infections have not been described in other susceptible decapods, particularly those where the parasite’s life cycle within the host is shortened, such as blue crabs where mortality occurs >35 days post exposure (Messick and Shields, 2000).

**Table 3.** Examples of effects of *Hematodinium* infection on physiological and behavioural processes

Effect	Crustacean	Comments	References
Respiration	*Nephrops norvegicus*	50% reduction in oxygen carrying capacity of haemolymph, x5 increase in oxygen consumption to compensate. Increase in anaerobic respiration	Taylor *et al.* (1996)
Moulting	*Cancer pagurus*	Increase in intermoult period following infection (i.e. suppression of moulting) but see Huchin-Mian *et al.* (2018) with no change in moult frequency in blue crabs	Smith and Rowley (2015)
Carbohydrate metabolism	*N. norvegicus* and *Callinectes sapidus*	Reduction in haemolymph glucose and hepatopancreatic glycogen with increasing severity of infection	Stentiford *et al.* (2001b); Shields *et al.* (2003)
Acid and alkaline phosphatases	*Portunus trituberculatus*	Short-term (3–96 hr) increase in enzyme activities in hepatopancreas of artificially infected crabs	Li *et al.* (2015a)
Protein	Various including *C. sapidus*	Reduction in haemolymph total protein including the respiratory pigment and immune factor, haemocyanin	Shields *et al.* (2003); Conneely and Coates (2025)
Muscle (meat) structure and biochemistry	*N. norvegicus*	Depletion of glycogen, alteration of amino acid profiles, some apparent structural damage and increased autolysis post-mortem affecting commercial value (and taste/meat texture)	Stentiford *et al.* (2000); Albalat *et al.* (2012)
Total haemocyte numbers	Various including *C. pagurus*	Reduction in haemocyte count (haemocytopenia) in later stages of infection probably resulting in heightened chance of secondary infections and reduced clotting	Smith and Rowley (2015); Rowley *et al.* (2015); Conneely and Coates (2025)
Behavioural	*C. sapidus*	Changes in behaviour including locomotion, burying and avoidance that may result in higher predation	Butler *et al.* (2014)

**Figure 4.**
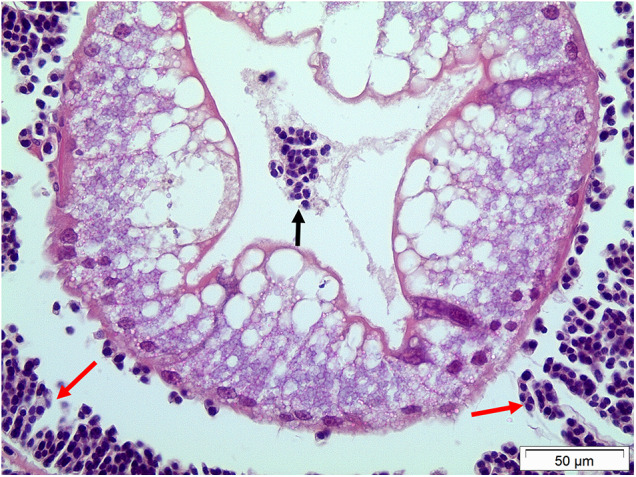
Histological section of the hepatopancreas of the shore crab, *Carcinus maenas* showing the presence of trophonts of *Hematodinium* sp. In the tubule lumen (unlabelled arrow). Note apparent integrity of the tubule cells and presence of filamentous plasmodia attached to the adjacent tubules (red arrows).

Histological studies only show modest changes in the integrity of affected tissues. Both trophonts and filamentous plasmodia are found in most tissues (Figure. 1A–D) simply because these are bathed in haemolymph. This latter form of the parasite is rarely seen in the haemolymph because it appears to be attached to cells in solid tissues including the gills (Figure. 1D) and hepatopancreas (Figure. 4). While subtle changes in muscle fibres have been reported (e.g. Stentiford *et al.*, 2000) these are modest and rarely observed histologically. The main change is the swelling of the spaces between the tubules of the hepatopancreas caused by the large increase in the number of circulating parasites but there is little evidence of their active penetration by the parasites although on rare occasions trophonts have been seen in the lumen of hepatopancreatic tubules (Figure. 4). Finally, changes in the gills including swelling and potential damage to the outer epithelia during dinospore proliferation in the gill lamellae have been observed (Wheeler *et al.*, 2007). Post-mortem changes such as cellular necrosis, particularly in the hepatopancreas, rapidly ensue following the death of hosts with accompanying bacterial multiplication in the cadavers (Smith and Rowley, 2015).

There is increasing evidence of the importance of dysbiotic changes in the microbiome during disease episodes in crustaceans (e.g. Bass *et al.*, 2019; Holt *et al.*, 2021; Hernández-Pérez *et al.*, 2022; Lorgen-Ritchie *et al.*, 2023). Changes in microbial diversity and community composition accompany diseases regardless of their causation. Examples include diseases of cultured shrimp such as those causing major economic effects highlighting the importance of determining how microbiomes change in response to disease (e.g. Boopathi *et al.*, 2023; Jatuyosporn *et al.*, 2023) and environmental change that potentiates disease (e.g. Cornejo-Granados *et al.*, 2018). In the case of parasitization caused by *Hematodinium* spp., both haemolymph and gut microbiomes are altered in the Norway lobster, *N. norvegicus* (Martin *et al.*, 2025). In lobsters with sub-patent infections (i.e. those that are PCR positive but negative using the less sensitive body colour and pleopod methods – see Table 2), bacterial species richness in the haemolymph is significantly reduced. The authors suggested that this effect could be significant in advancing the susceptibility to secondary infections in such animals. Furthermore, as microbial dysbiosis has been found to result in changes to immune competence in several disease conditions in crustaceans (e.g. Zhou *et al.*, 2025) this may be of importance in this disease.

## The crustacean immune system – a brief overview

This section aims to give a brief overview of how crustaceans deal with infectious agents such as *Hematodinium* spp. that penetrate their internal tissues with reference to how they defend against such micro-parasites. Because this is purposely a succinct overview, the reader is also referred to several recent reviews for greater detail (e.g. Coates *et al.*, 2022; Rowley, 2026).

The cells at the centre of the immune system of crustaceans are the circulating blood cells termed haemocytes. These cells are formed in discreet haemopoietic tissues (Söderhäll and Söderhäll, 2022). There are 3 main types of haemocytes in crustaceans, namely hyaline cells, semi-granular haemocytes (= semi-granulocytes) and granular haemocytes (= granulocytes). These 3 types are morphologically distinct based on the number and size of granular inclusions in the cytoplasm. Each cell type has a distinct role in defence and haemostasis including phagocytosis, nodule/capsule formation, coagulation and wound healing. For instance, hyaline cells are actively phagocytic and are found in the flattened sheath of cells surrounding nodules (Rowley, 2026) while granular haemocytes are highly unstable and quickly respond to injury by bringing about blood clotting and the triggering of the prophenoloxidase activating system. Recent approaches looking at the biological properties of individual haemocytes have revealed further diversity in these 3 cell types (Söderhäll *et al.*, 2022; Xin and Zhang, 2023; Cui *et al.*, 2024) suggesting the presence of a series of sub-populations or different stages in the ontogeny of such cells.

Crustacean haemocytes, together with other cell types, also synthesize a range of immune-active molecules involved in the recognition and killing of extra- and intra-cellular pathogens and parasites. These include factors produced by the prophenoloxidase activating system (Cerenius and Söderhäll, 2021), various lectins (Sanchez-Salgado *et al.*, 2017) and antimicrobial peptides including crustins (Barreto *et al.*, 2022) and penaeidins (Destoumieux *et al.*, 2000) to name just a few. A terminal product of the prophenoloxidase activating system is the dark pigment, melanin, that is a useful marker of immune reactivity seen following cuticular damage and in the nodules and capsules surrounding parasites, pathogens and damaged tissues within the haemocoel.

Of importance to our understanding of the interaction between host and its parasites and pathogens, is how the crustacean immune system deals with these insults. For example, injection of microbes into the haemocoel of shore crabs (*Carcinus maenas*) results in their rapid clearance from circulation by a combination of phagocytosis and nodule formation (Smith and Ratcliffe, 1980). This latter process forms nodules that consist of a melanized core containing the invaders together with the remnants of degranulated granular haemocytes surrounded by a multicellular flattened sheath of hyaline and semi-granular cells (Smith and Ratcliffe, 1980; Ratcliffe *et al.*, 1985). In the case of microbial pathogens of crabs, including some fungi, they are sequestered into nodules (i.e. recognized and dealt with as ‘foreign’) but they are resistant to the killing mechanisms, and they multiply within the melanotic cores and in the phagosomes of haemocytes, to escape and cause a fatal septicaemia (Smith *et al.*, 2013). Non-pathogens and avirulent strains of pathogens are usually sequestered within nodules and their soluble breakdown products stored within nephrocytes in gills (Smith and Ratcliffe, 1981). While the methods of recognition of microbial agents such as viruses, bacteria and fungi are known, the equivalent process with eukaryotic micro- and macro-parasites has not been studied. Similarly, knowledge of how the immune system kills or inhibits the development of micro- and macro-parasites is wanting.

## How do *Hematodinium* spp. avoid or circumvent the immune response of crustaceans?

A fundamental observation made from examining haemolymph preparations and tissues via histology is that *Hematodinium* is neither phagocytosed nor incorporated into nodules (Rowley *et al.*, 2015; Smith *et al.*, 2015; Davies *et al.*, 2019, 2022; Martin *et al.*, 2025) and this supports the concept that this parasite is not recognized as ‘foreign’ by the crustacean immune system (Rowley *et al.*, 2015).

There are several ways in which parasites and pathogens can inhibit and/or avoid the immune response of animals, so that they can invade, reproduce or reside in their tissues unmolested. These include:

1. Mimicking host tissues and hence being ‘invisible’ to the immune system (i.e. they do not appear to elicit a cellular immune response)
2. Residing in locations that avoid immune activation (e.g. ‘hiding’ inside host cells)
3. Having mechanisms that defeat the immune response allowing them to replicate in host tissues (e.g. pathogenic fungal infections of crabs [e.g. Smith *et al.*, 2013] as already described)

### Molecular mimicry

Molecular mimicry is a mechanism utilized by parasites and pathogens, such as viruses, to avoid eliciting an immune response. They achieve this by displaying antigens on their surfaces that resemble those of the host and hence they do not either elicit an immune response or only a limited response ensues. Examples of this phenomenon that have received in-depth study within invertebrates are few, but a notable exception is how the parasitic helminth, *Schistosoma mansoni*, survives during its development in the molluscan intermediate host, *Biomphalaria glabrata* (see Hambrook and Hanington, 2021 for a review). By using resistant and susceptible strains of *B. glabrata,* it has been possible to tease-out the determinants that influence the interaction between host and parasite. These approaches have identified several mechanisms utilized by different stages of the parasite while associated with snails. These include molecules containing glycan epitopes shared between the host’s haemocytes and larval schistosomes (Yoshino *et al.*, 2013). It should be stressed, however, that schistosomes have other mechanisms to circumvent/suppress the host’s immune system showing that molecular mimicry alone does not fully explain how these parasites survive when in their molluscan host (Hambrook and Hanington, 2021). Extrapolating from this host-parasite interaction, it is possible that *Hematodinium* and host haemocytes of susceptible crustaceans share common antigens but to date, this has not been explored. It would also seem likely that these parasites rely on a variety of methods other than just molecular mimicry.

### Other potential mechanisms of immune suppression

Although observations fail to reveal a cellular (i.e. phagocytosis or nodule formation) response in both natural and experimental infections with *Hematodinium*, several studies suggest these parasites employ a variety of immune suppressive activities affecting the generation of recognition and killing factors (Li *et al.*, 2015a, 2016, 2018, 2022). These observations have been made in the Chinese swimming (= gazami) crab, *P. trituberculatus* following its artificial infection with trophonts of *H. perezi* genotype II. The dynamics of these changes appear to indicate a short term, rather than a sustained effect post-challenge, and different investigative approaches utilized by these authors have given contradictory results. For example, phenoloxidase enzyme activity in haemocytes, as measured spectrophotometrically, has been found to rapidly decline over a 16-day period post-challenge with *Hematodinium* (Li *et al.*, 2022) but the gene expression of prophenoloxidase (*proPO*) over an 8 day period in the same host shows both elevation and inhibition at different time periods (Li *et al.*, 2015b), and the same enzyme activity in the hepatopancreas is consistently raised from 3 hr to 8 days post-infection (Li *et al.*, 2015a). Whether the products of the phenoloxidase activating system interact with this parasite is also unknown and so these contradictory observations may not be of direct relevance to its development in the host. Similarly, Li *et al.*, (2016) reported changes in the enzyme, nitric oxide synthase, post-challenge with *Hematodinium*. The product of this enzyme, nitric oxide, is an important factor in the intracellular killing of parasites and pathogens within professional phagocytes, but as *Hematodinium* spp. do not reside within such cells, it is unclear what significance this could have in the pathogenesis of this parasite.

## *Exosomes and crustacean diseases including those caused by* Hematodinium

Some recent developments may provide a new approach to study the interaction of parasites and pathogens including *Hematodinium* spp. and their crustacean hosts. These have investigated the potential role of extracellular vesicles (e.g. exosomes and microvesicles) in the pathogenesis of various diseases. Extracellular vesicles are heterogeneous in size but generally small (>200 nm) membrane-bound structures that can transport proteins (e.g. growth factors, enzymes), lipids and nucleic acids (DNA, mRNA and miRNA) between host cells, and pathogen/parasite and host. They are produced by a wide range of organisms including animals, plants and bacteria and play a role in intercellular communication (Liu and Wang, 2023) and host-disease interactions (Shetty and Upadhya, 2021; Yates *et al.*, 2022). One of the first reports showing the importance of exosomes in crustacean diseases came from an investigation of tremor disease caused by *Spiroplasma eriocheris* (Ma *et al.*, 2024). This is an important pathogen of freshwater crustaceans including the Chinese mitten crab, *Eriocheir sinensis* (Wang *et al.*, 2004; Coates and Rowley, 2022). *S. eriocheris* is an intracellular pathogen that can develop within the host’s haemocytes resulting in death. Ma *et al.* (2024) found that exosomes released by haemocytes from spiroplasma-infected crabs contained immune-active molecules including prophenoloxidase, lectins and tetraspasnin. These exosomes were found to be important in aiding the host defences of crabs by inducing the phagocytic and apoptotic activities of haemocytes and the tetraspasnin cargo of these exosomes appears to be of importance in this protective activity.

Two recent studies have investigated the potential role of exosomes in the parasitization of crabs by *Hematodinium* spp. (Coates *et al.*, 2023; Zhang *et al.*, 2025a). Coates *et al.* (2023) found a reduction in the number of extracellular vesicles in the haemolymph of shore crabs (*C. maenas*) naturally infected with *Hematodinium* sp. (probably *H. perezi* genotype I). Their study also observed the post-translational modification of immune factors in infected crabs by deamination including actin, non-inducible nitric oxide synthase and the tail-less form of Down syndrome cellular adhesion molecule. This latter molecule has been identified as a multivariant recognition factor that is involved in the internalization of various microbes by professional phagocytes in arthropods including crustaceans (Ng and Kurtz, 2020; Rowley, 2026) but its potential interaction with *Hematodinium* is unknown. The second of these studies gives further and detailed insight into how exosomes may influence the immune response to *Hematodinium* (Zhang *et al.*, 2025a). This study observed that *H. perezi* genotype II release exosomes that come to reside in the peri-nuclear region of the host’s (*P. trituberculatus*) haemocytes. The cargo of these exosomes includes miRNAs that suppress part of the Toll pathway that is important in the recognition of some bacteria and fungi resulting in their internalization by haemocytes and switching on the production of antimicrobial peptides (Habib and Zhang, 2020; Rowley, 2026). Of note was the finding that the *in vitro* phagocytosis of test bacteria (*E. coli*) by haemocytes from *Hematodinium* infected crabs was inhibited and this was linked to the gene *rictor* that is a target of the parasite-delivered miRNAs (Zhang *et al.*, 2025a). While it should be remembered that *Hematodinium* spp. are not phagocytosed by crustacean haemocytes, this inhibition could leave such animals with reduced ability to deal with secondary infections caused by bacteria and some fungi.

### Are there explanations of contrasting viewpoints on how *Hematodinium* circumvents the host’s immune system?

There are potentially conflicting viewpoints of how *Hematodinium* spp. survive and replicate in the haemolymph of susceptible hosts apparently unmolested. For instance, Rowley *et al.*, (2015) showed that the dynamics of bacterial clearance (a marker of phagocytosis and nodule formation) in *Hematodinium* infected *vs*. non-infected edible crabs did not differ. This implies a mechanism of action that does not compromise how the immune system deals with other invaders (i.e. general immune suppression). However, others have demonstrated changes in the antimicrobial armoury and recognition pathways in *Hematodinium* infected crabs (Li *et al.*, 2015a, 2015b, 2016, 2018, 2022) that would be expected to result in a reduction in the ability of such animals to remove any invaders from circulation. These apparent differences may be explained by the diversity of model hosts, parasite genotypes and the severity of infections in these studies. Rowley *et al.*, (2015) used edible crabs (*C. pagurus*) in their study while others (Li *et al.*, 2015a, 2016, 2018, 2022) made use of the Chinese swimming crab, *P. trituberculatus* as a model species where the dynamics of infection with *Hematodinium* spp. differ. Depending on the genotype investigated, studies on parasite and haemocyte number both in naturally and artificially infected edible crabs reveal that the replication of the parasite is initially very slow, especially in genotype I, and that it is only in the late phase several months post-infection when haemocyte numbers rapidly decline and trophonts mass in the haemolymph (Figure. 3). The preliminary experiments described in Rowley *et al.*, (2015) and Smith and Rowley (2015) used crabs with only modest infections where the haemocyte numbers were unaffected by parasitization. Clearly, when the haemocyte numbers decline in late-stage infections this must leave crustaceans vulnerable to secondary infections such as those caused by fungi (Smith *et al.*, 2013). In *P. trituberculatus*, however, post-challenge with trophonts of *H. perezi* genotype II, there is a rapid increase in parasite number over just a few days with ensuing mortality and the rapid changes in the capacity of the immune system in terms of haemocyte number alone would be more pressing.

Taken together, these studies with both *C. pagurus* and *P. trituberculatus* probably demonstrate that *Hematodinium* spp. (i.e. *H. perezi* genotypes I and II respectively) are utilizing a range of mechanisms to allow these parasites to develop unmolested in the tissues but there is still uncertainty about whether molecular mimicry plays an important role in these events and the relevance of immune molecules such as phenoloxidase that produce products that may not directly or indirectly kill these parasites. While the histopathology of infections caused by genotypes I–III are all remarkably similar, the dynamics and outcomes of infections appear to differ markedly. For instance, the aquarium-based infection studies of edible crabs (*C. pagurus*) by Smith and Rowley (2015) imply that natural infections in cool waters in northern Europe by this genotype of *H. perezi* (genotype I?) may take several months to establish themselves and that at least some of the mortality observed is caused by other infections rather than from this parasite alone. In contrast, crustaceans in warmer waters, such as *P. trituberculatus* infected with *H. perezi* genotype II in China, succumb to rapidly developing infections (Li *et al.*, 2021b) apparently without the participation of other infectious agents. Indeed, the same genotype of *H. perezi* (II), can cause death of mudflat crabs (*H. tientsinensis*) in only 4 weeks post-challenge (Zhang *et al.*, 2025b). It would be interesting to determine if the outcome of such infections is attributable to differences in the 3 genotypes of *H. perezi*. For instance, if susceptible crabs held in standardized temperature conditions are artificially infected with each of the different genotypes does the speed and outcome of such challenges differ? This would answer the relative importance of genetic makeup of the parasite *vs*. environmental and/or host parameters in determining the outcome of infections and interaction with the host’s immune system.

## Conclusions and future directions

In the ensuing century since the initial discovery of *Hematodinium* in northern Europe, this endoparasite has been recognized as a serious threat to many species of decapod crustaceans as it has been found to have resulted in high levels of prevalence in some populations. The recent observation of *H. perezi* genotype II in crabs under cultivation in polyculture systems in parts of China, where reservoirs of infection can occur in adjacent wild crabs, is of concern because such systems have a poor prospect of applying any of the principles of biosecurity to limit their spread. Commercially fished populations, including Tanner and snow crabs in cold waters in north Americas, are also at future risk with changes in the sea water temperature particularly in the winter months that can limit the development of these parasites.

There have been significant advances in the understanding of the effects of this parasite on its hosts and the methods that allow the accurate identification of *Hematodinium* spp. but key questions remain unanswered. These include:

1. Are all hosts killed by infections caused by *Hematodinium* spp.? (i.e. are there latent infections as considered by Eigmann *et al.*, 2010?).
2. Do the 3 genotypes of *H. perezi* have differing infection dynamics and severity resulting in varying rates of mortality? (i.e. are some genotypes more virulent than others regardless of water temperature?).
3. Why are some species of homarids (clawed lobsters) apparently not naturally infected by *Hematodinium* spp.?
4. Do the 3 genotypes (I–III) of *H. perezi* each have distinct host ranges that are independent of geographic location?
5. What risks will change in sea water temperature in the future pose to the viability of some commercial fisheries in the presence of some parasites such as *Hematodinium* spp.?
6. Is molecular mimicry a driver of the success of these parasites facilitating their multiplication in the host’s tissues and an explanation of their wide host range?
7. How important are parasite-derived exosomes in the development of *Hematodinium* spp. in their hosts?
8. How do dinospores of *Hematodinium* spp. gain entry to the tissues of their hosts? and
9. What are the triggers and mechanism(s) for parasite release from the host?
